# The Effects of Proteasome Inhibitors on Telomerase Activity and Regulation in Multiple Myeloma Cells

**DOI:** 10.3390/ijms20102509

**Published:** 2019-05-21

**Authors:** Naama Shalem-Cohavi, Einat Beery, Jardena Nordenberg, Uri Rozovski, Pia Raanani, Meir Lahav, Orit Uziel

**Affiliations:** 1 The Felsenstein Medical Research Center, Beilinson Medical Center; Petah-Tikvah 49100, Israel; Naama.shalem@gmail.com (N.S.-C.); einatb@clalit.org.il (E.B.); Jardenan@tauex.tau.ac.il (J.N.); rozovski.uri@gmail.com (U.R.); piar@clalit.org.il (P.R.); mlahav@tauex.tau.ac.il (M.L.); 2 Hematology Institute, Davidoff Cancer Center, Rabin Medical Center Petah–Tikva and Sackler School of Medicine, Tel-Aviv University, Ramat-Aviv 69978, Israel

**Keywords:** proteasome inhibitors, telomerase, multiple myeloma cells

## Abstract

The importance of telomerase, the enzyme that maintains telomere length, has been reported in many malignancies in general and in multiple myeloma (MM) in particular. Proteasome inhibitors are clinically used to combat effectively MM. Since the mechanism of action of proteasome inhibitors has not been fully described we sought to clarify its potential effect on telomerase activity (TA) in MM cells. Previously we showed that the first generation proteasome inhibitor bortezomib (Brt) inhibits TA in MM cells by both transcriptional and post-translational mechanisms and has a potential clinical significance. In the current study we focused around the anti- telomerase activity of the new generation of proteasome inhibitors, epoxomicin (EP) and MG-132 in order to clarify whether telomerase inhibition represents a class effect. We have exposed MM cell lines, ARP-1, CAG, RPMI 8226 and U266 to EP or MG and the following parameters were assessed: viability; TA, hTERT expression, the binding of hTERT (human telomerase reverse transcriptase) transcription factors and post-translational modifications. Epoxomicin and MG-132 differentially downregulated the proliferation and TA in all MM cell lines. The downregulation of TA and the expression of hTERT were faster in CAG than in ARP-1 cells. Epoxomicin was more potent than MG-132 and therefore further mechanistic studies were performed using this compound. The inhibition of TA was mainly transcriptionally regulated. The binding of three positive regulator transcription factors: SP1, c-Myc and NF-κB to the hTERT promoter was decreased by EP in CAG cells as well as their total cellular expression. In ARP-1 cells the SP1 and c-MYC binding and protein levels were similarly affected by EP while NF-κB was not affected. Interestingly, the transcription factor WT-1 (Wilms’ tumor-1) exhibited an increased binding to the hTERT promoter while its total cellular amount remained unchanged. Our results combined with our previous study of bortezomib define telomerase as a general target for proteasome inhibitors. The inhibitory effect of TA is exerted by several regulatory levels, transcriptional and post translational. SP1, C-Myc and NF-κB were involved in mediating these effects. A novel finding of this study is the role of WT-1 in the regulation of telomerase which appears as a negative regulator of hTERT expression. The results of this study may contribute to future development of telomerase inhibition as a therapeutic modality in MM.

## 1. Introduction

Multiple myeloma (MM) originates from B-lymphocytes which are transformed into plasma cells that accumulate in the bone marrow, secreting monoclonal immunoglobulin and damage the bone tissue. MM consists of 1% of all cancers and 10% of hematological malignancies. It is considered as the malignancy with the worst prognosis and lowest survival rates [1,2]. A relatively new treatment in MM is the proteasome inhibitors Bortezomib [3]. Although efficient, Bortezomib treatment may result in the development of resistance against this drug. Therefore, the search for novel drugs is continuously ongoing. The proteasome system controls the degradation of most cellular proteins [4]. This system plays a crucial role in processes such as proliferation, cell cycle, DNA damage repair, response to stress and apoptosis [5]. In addition, the proteasome machinery regulates the level of NF-κB, a transcription factor mediates the expression of genes related to proliferation, apoptosis and adhesion [6,7]. This explains the nature of the proteasome as a valid anticancer drug target. Inhibition of the proteasome results in decreased tumor expansion, increased apoptosis, inhibition of metastasis and angiogenesis [8] with reduced cytotoxicity to normal cells [9]. Bortezomib is the first proteasome inhibitor that is in active clinical use in MM [10]. Although usually treatment with Bortezomib is efficient, in numerous cases there was no response or resistance to the drug [11,12]. MG-132 is a peptide aldehyde which inhibits the proteasome as well as other proteases in vitro [9]. Epoxomicin is another inhibitor of the proteasome which covalently attaches to the b sub-unit of the proteasome in an irreversible manner [13]. Carfilzomib is an EP analogue which is much more potent as an anti-MM drug, especially on the resistant ones [14,15].

The importance of telomerase in the biology and prognosis of many types of cancers including MCL is well established [16]. Telomerase is a unique reverse transcriptase expressed almost exclusively in >90% of cancer cells. It compensates for telomeric loss in each DNA replication [17] thus conferring endless replicative potential to the cancer cell. Due to its essentiality and specificity to the malignant cell it may serve as a valid anticancer drug target and indeed active compounds that target telomerase are already in advanced phases of clinical trials [18]. The importance of telomerase in MM has been demonstrated convincingly both in vitro and clinically. Numerous cytotoxic drugs target telomerase [19,20,21]. We also showed that Bortezomib downregulates telomerase activity in myeloma cells and this inhibition may have clinical implications [22]. In the light of the inhibitory effect of Bortezomib on MM cells we surmised that the analogue of the new generation of proteasome inhibitors, EP, may be more potent in inhibition of the activity of telomerase in MM cells. In the current paper we present data regarding the effect of epoxomicin and MG-132 on telomerase activity in MM cells and analyze the regulatory pathways leading to this inhibitory effect.

## 2. Results

### 2.1. The Effects of Epoxomicin and MG-132 and on the Proliferation of MM Cells

**Figure 1:**
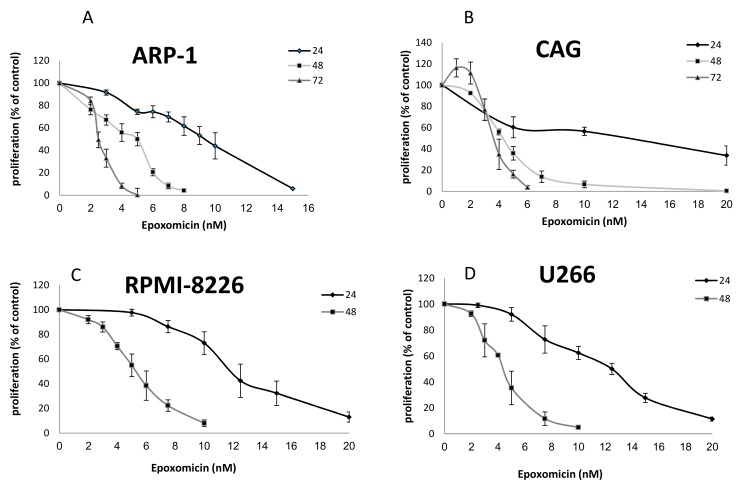
Proliferation of multiply myeloma (MM) cells in response to epoxomicin treatment. MM cells were grown in the presence of a range of epoxomicin dosages and their proliferation was assessed by the WST-1 assay. (**A**) ARP-1 cells; (**B**) CAG cells; (**C**) RPMI-8226 cells; (**D**) U266 cells.

**Figure 2:**
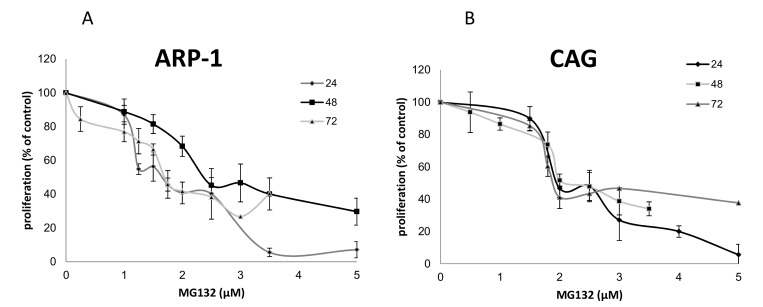
Proliferation of MM cells in response to MG-132 treatment. MM cells were grown in the presence of a range of MG-132 dosages and their proliferation was assessed by the WST-1 assay. (**A**) ARP-1 cells; (**B**) CAG cells.

The proliferation of CAG, ARP-1, U266 and RPMI-8226 cells was monitored after the administration of MG-132 or epoxomicin for 24, 48 and 72 h. These exposure times were selected based on our previous experiences with another proteasome inhibitor, Bortezomib [23]. Both drugs inhibited cell proliferation in a dose dependent manner in all cell lines (Figure 1A–D). The inhibitory effect of epoxomicin that was examined in the above four MM cells found to be similar in all cells and was time dependent with an IC_50_ of 2–10 nM for the ARP-1 cells, 4–12 nM for the CAG cells, 4–13 nM for the U266 cells and 2–10 nM for the RPMI8226 cells. However, the inhibitory effects of MG-132 were different and much less pronounced (three orders of magnitude): the time of exposure did not affect the inhibition of growth. For the ARP-1 cells, an IC_50_ of about 1.8–2.5 µM was detected and for the CAG cells, an IC_50_ of about 2 µM was calculated. Since these effects were similar to the effects obtained for U266 and RPMI-8226 [24], ARP-1 and CAG cells were chosen as representative MM models for studying the effect of MG-132 on MM cells (Figure 2A,B).

### 2.2. TA Following Treatment of MM Cell Lines by Epoxomicin

**Figure 3:**
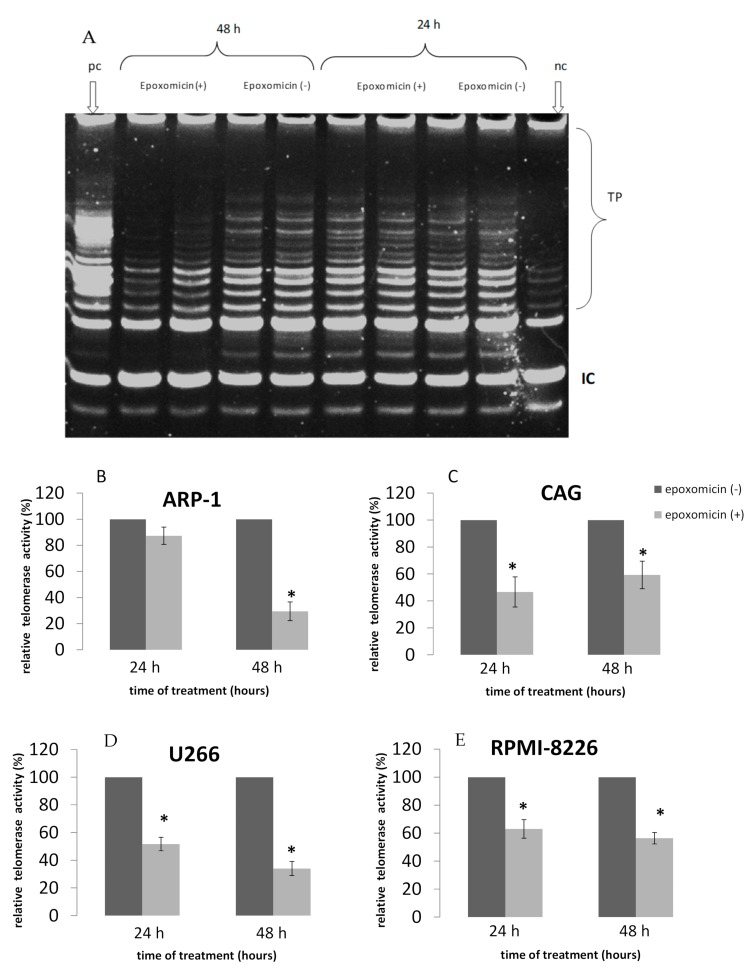
TA of MM cells in response to epoxomicin. MM cells were grown in the presence of the IC_50_ of epoxomicin for 24 and 48 h and telomerase activity was assessed by the TRAP assay. (**A**) A representative example of the TRAP assay. TP—telomerase products; IC—internal PCR control; PC—positive control (a mixture of six templates competent for amplification by the primers of the TRAP assay); NC—negative control—no telomerase extract in the reaction. (**B**) ARP-1 cells; (**C**) CAG cells; (**D**) U266 cells; (**E**) RPMI-8226 cells. * denotes statistical significance (*p* < 0.05).

After inspecting the inhibitory effect of epoxomicin on cell proliferation we wanted to assess its putative effect on telomerase activity, to understand whether the decrease in cell proliferation is mediated by or accompanied with a reduction in telomerase activity of the cells. TA was assessed in all MM cells after 24 h and 48 h exposure to the above IC_50_ concentrations. All MM cells exhibited a differential decrease in TA in response to epoxomicin exposure. In ARP-1 cells a decrease of 70% of TA was obtained (*p* = 0.007) after 48 h of epoxomicin exposure. TA decreased in CAG cells 24 h after the drug exposure by 55% (*p* = 0.021), which stayed about the same after 48 h of exposure, suggesting that CAG cells are less sensitive to the drug. U266 cells responded to the treatment after 24 h of exposure, while after 48 h a decrease of 63% was obtained (*p* = 0.022). RPMI-8226 cells decreased TA in response to the drug treatment in a similar way to U266 24 and 48 h after treatment (Figure 3). 

### 2.3. TA Following Treatment of MM Cell Lines by MG-132

**Figure 4:**
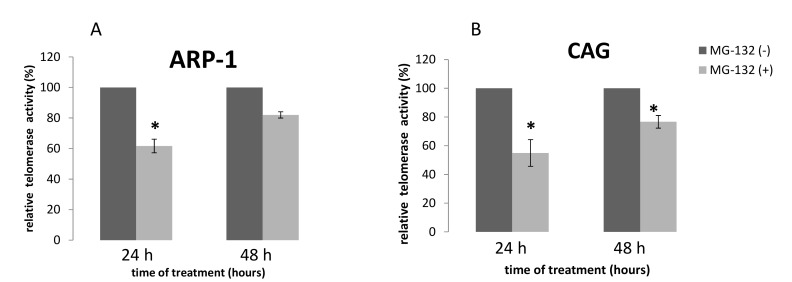
TA of MM cells in response to MG-132. MM cells were grown in the presence of the IC_50_ of epoxomicin for 24 and 48 h and telomerase activity was assessed by the TRAP assay. (**A**) ARP-1 cells; (**B**) CAG cells; * denotes statistical significance (*p* < 0.05).

Like epoxomicin, MG-132 affected TA, but in a different mode. The effect of the drug on both cell lines: CAG and ARP-1 was similar. In addition, the inhibitory effect of MG-132 on TA diminished with time: about 40% decrease after 24 h that was reduced to 20% 48 h post exposure to the drug (Figure 4).

### 2.4. The Effect of Epoxomicin on Telomerase Regulation

In order to decipher the mechanism underlying the effect of proteasome inhibitors on telomerase regulation, epoxomicin was chosen due to its greater impact on the activity of telomerase compared to MG-132. We studied the relevant mechanism in two representative cell lines: ARP-1 and CAG. Two levels of telomerase regulation, both considered as the major regulatory modalities, were explored: the transcriptional level and the post translational one. 

### 2.5. The Effect of Epoxomicin on the Transcription of the hTERT Gene

**Figure 5:**
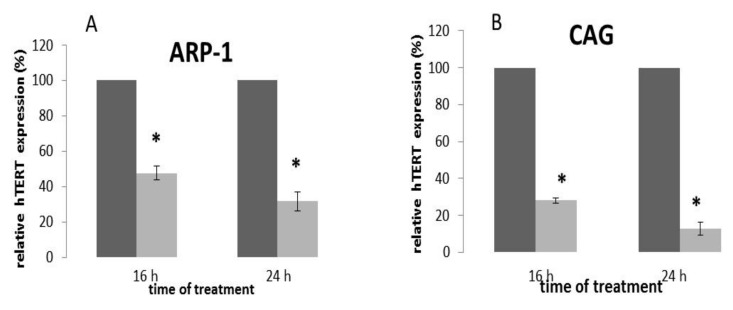
The transcriptional effect of epoxomicin on the hTERT gene expression. MM cells were grown in the presence of the IC_50_ of epoxomicin for 24 and 48 h and the expression of the hTERT gene was assessed by real time PCR. (**A**) ARP-1 cells; (**B**) CAG cells; * denotes statistical significance (*p* < 0.05).

To decipher whether the transcription level of the hTERT gene was changed in response to epoxomicin treatment we measured the mRNA levels of hTERT after epoxomicin treatment. As shown in Figure 5, both cell lines exhibited a major decrease in the transcript levels of hTERT in response to the drug. In CAG cells, this transcript was downregulated in about 70% 16 h post treatment as opposed to a decrease of 50% in the ARP-1 cells. This goes in line with the decrease in TA obtained in these cells 24 h post drug treatment.

### 2.6. The Effect of Epoxomicin on the Post-Translational Modification of Telomerase

**Figure 6:**
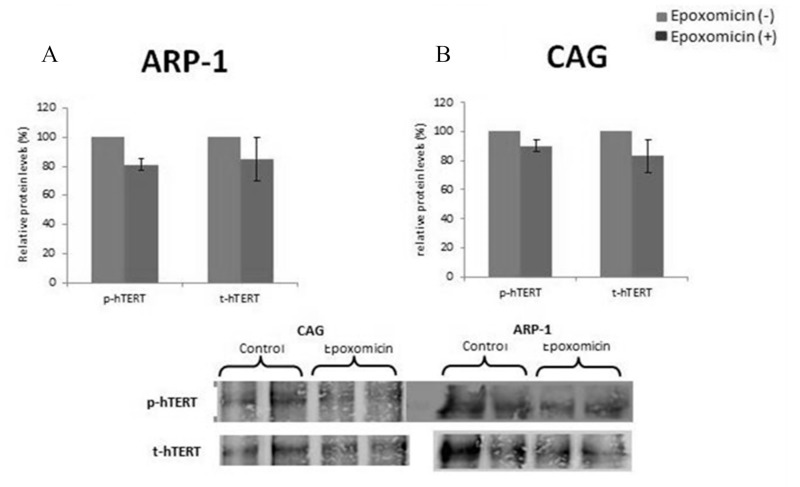
The post-translational effect of epoxomicin on telomerase phosphorylation. MM cells were grown in the presence of the IC_50_ of epoxomicin for 24 and 48 h and the phosphorylation status of telomerase was measured by Western blot. (**A**) ARP-1 cells; (**B**) CAG cells; * denotes statistical significance (*p* < 0.05). On the lower panel an example of the Western blot is shown.

We assessed the level of telomerase phosphorylation after the various treatments. The results showed that no significant change of telomerase phosphorylation was observed in response to the drug exposure, suggesting that the regulation of telomerase in this setting is not post-translational (Figure 6).

### 2.7. The Effect of Epoxomicin on the Cellular Levels and Promoter Binding of Several Transcription Factors of the Core Promoter of Telomerase

**Figure 7:**
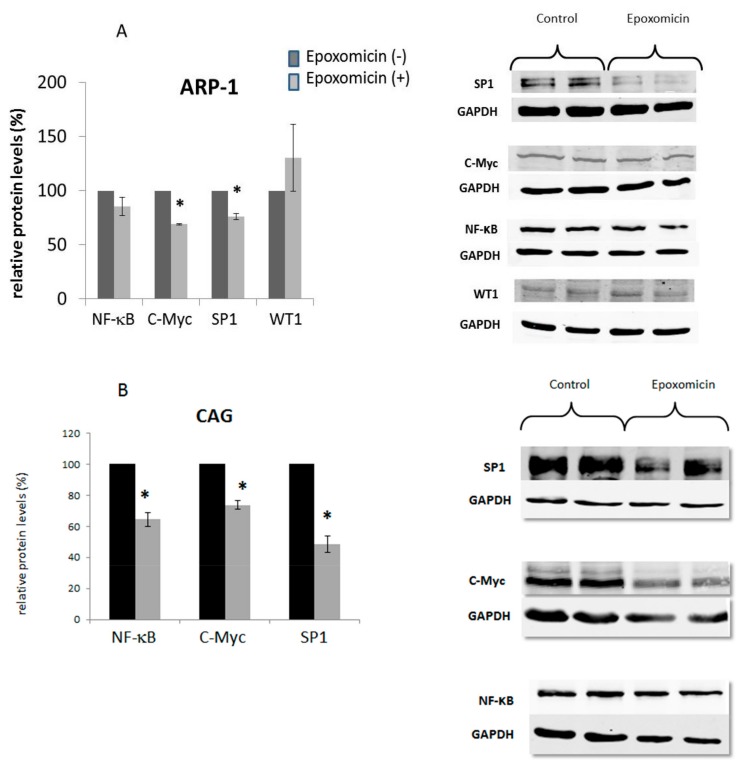
The effect of epoxomicin on the cellular levels of the hTERT transcription factors. MM cells were grown in the presence of the IC_50_ of epoxomicin for 24 and 48 h and the levels of telomerase transcription factors were measured by Western blot. (**A**) ARP-1 cells; (**B**) CAG cells; * denotes statistical significance (*p* < 0.05). On the right panel examples of the Western blot are shown.

Since the expression of hTERT was highly affected by epoxomicin, we wanted to find whether the binding of its major transcription factors was damaged in response to the drug. Prior to that assay we monitored the cellular levels of these factors by Western blotting. The results showed that in CAG cells the three transcription factors of telomerase decreased after epoxomicin treatment: NF-κB, C-Myc and SP-1 levels decreased in 40%, 30% and 55%, respectively (Figure 7). In ARP-1 cells these factors levels decreased in 30% and 25% for C-Myc and SP-1 while NF-κB levels did not change in response to epoxomicin (Figure 7). The levels of the transcription factor WT-1 in ARP and CAG cells did not change in response to the drug. 

**Figure 8:**
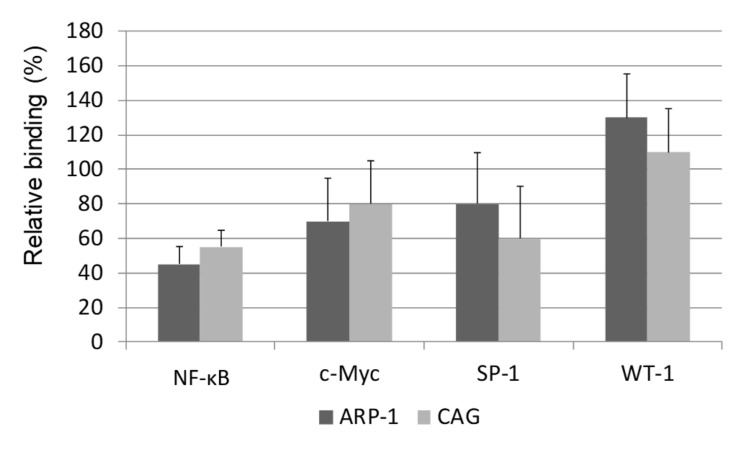
The effect of epoxomicin on the binding of the hTERT transcription factors to its promoter. ARP-1 and CAG cells were grown in the presence of the IC_50_ of epoxomicin for 24 and 48 h and the levels of the binding of telomerase transcription factors to its core promoter were measured by the ChIP assay.

We then assessed the level of the binding of these transcription factors to the hTERT promoter by the ChIP assay. As shown in Figure 8, all three positive factors: C-Myc, SP-1 and NF-κB decreased their binding to the hTERT promoter after the drug exposure. In contrast, the WT-1 transcription factor increased its binding to the hTERT promoter in these setting (Figure 8).

## 3. Discussion

Telomerase, the hallmark of cancer, is considered a valid anticancer drug target since it meets the two criteria defining an appropriate drug target: specificity (specific mainly to cancer cells) and essentiality (telomerase confers limitless life span to cancer cells). In the current study we show for the first time that telomerase is a target for proteasome inhibitors MG-132 and epoxomicin.

Our results demonstrate a differential ability of both drugs to inhibit proliferation and TA in MM cells by decreasing the *hTERT* mRNA expression. Epoxomicin was more potent than MG132 in this regard. Epoxomicin’s effect on the proliferation of the cells was time and dose specific, whereas the effect of MG-132 was similar at different time exposures. This difference probably stems from the differences in their mechanism of action. Epoxomicin is an irreversible inhibitor that covalently bound to the proteasome, therefore its effect is enhanced with time. In contrast, MG-132 is a reversible inhibitor, competing with the other natural substrates of the proteasome until reaching a homeostasis. Therefore, longer incubation time with this drug does not enhance its effect. MM cells exhibited differential sensitivity to the anti-proliferative effect induced by both inhibitors. CAG cells were found to exhibit a higher sensitivity to the drug than ARP-1 cells. However, epoxomicin effects on all four tested MM cells were more or less similar. In a previous study conducted in our lab we have reported that Bortezomib, another proteasome inhibitor, inhibits the proliferation (and TA) in MM cells with a similar IC50 dosage [22]. In a similar study we have found that Bortezomib affects the proliferation (and TA) in mantel cell lymphoma [23]. The effect on the proliferation of the cells was reported in several other studies as well [25].

Epoxomicin was able to inhibit telomerase activity in all four tested cell lines albeit in a different kinetics. CAG cells exhibited a quicker response to the drug, as 24 h of drug exposure was enough to reduce TA by 55% that reached 40% after 48 h of epoxomicin exposure. In ARP-1 cells, TA was reduced by 70% after 48 h of epoxomicin exposure. U266 and RPMI 8226 responded similarly to epoxomicin, as TA was reduced to ~50% 24 h after the drug exposure. These differences may be attributed to the differences in the genetic makeup of the cells and or differences in their membrane permeability. In contrast to epoxomicin, MG-132 reduced TA in a similar in all MM cells. TA in all tested cells was reduced by 40% and 20% 24 and 48 h respectively after MG-132 exposure, possibly due to the mechanistic differences in the drugs activities, as MG-132 is a reversible inhibitor and therefore its effect reduced with time. 

TA is regulated at multiple levels, including transcription, mRNA splicing, maturation and modifications of hTR and hTERT, transport and sub cellular localization of each component, assembly of the holoenzyme to an active ribonucleoprotein, accessibility and proper function on its telomeric substrates [26]. To understand the mechanism by which epoxomicin inhibits TA in the cells we assessed the effect of epoxomicin, the more potent and clinically used drug (Carfilzomib), both on the expression of the hTERT and on the post-translational level in MM cells. A marked decrease in the expression of hTERT was obtained in both MM cell lines in response to epoxomicin treatment. The transcript in CAG cells decreased by 70% 16 h post treatment while in ARP-1 cells the decrease was of 50%. This difference is in line with the decrease in TA in these cells 24 h post treatment. 

Previous studies showed that TERT promoter activity is usually regulated by a variety of transcription factors like: AP-1, NF-κB, c-Myc, SP1 and the estrogen receptor [27,28]. We assessed the levels of four transcription factors, C-Myc, SP-1, NF-κB and WT-1, binding to the hTERT promoter in response to epoxomicin exposure in order to understand how the drug decreases the expression of the hTERT gene. The levels of three transcription factors binding to the hTERT promoter, C-Myc, SP-1, NF-κB, were reduced after the drug exposure. Partially, this decrease is due to a total decrease of these proteins in response to epoxomicin. This suggests that the decrease in the expression of the hTERT gene after epoxomicin is controlled by the decrease in the transcription factors binding to the hTERT promoter. In contrast, WT-1 increased its binding to the hTERT promoter after the drug exposure, defining it as a possible negative regulator of TA. The identity of WT-1 with regards to the expression of the hTERT is controversial [29]. One study claimed that the identity of this transcription factor is dependent on cell type [30]. Previous studies found that WT-1 is indeed a negative regulator of TA [31]. In kidney cells it acted as a negative regulator of the hTERT [32]. As opposed to somatic cells, in cancer cells WT-1 was shown to act as a negative regulator of hTERT [32]. 

Epoxomicin did not change the levels of phosphorylation of telomerase, suggesting that the regulation on TA in this setting is not mediated by post-translational level.

The phenotype of a given cell after exposure to the proteasome inhibitor results from the effects of many signal transduction processes that are either inhibited or catalyzed by that treatment. Therefore, we cannot exclude the possibility that additional routes may be involved in the regulation of telomerase inhibition and therefore other intra-cellular mechanism should be examined in these settings.

Similar studies conducted in our lab have shown that Bortezomib, another inhibitor of the proteosome, decrease proliferation and TA in MM and mantle cell lymphoma cells, albeit via different mechanisms [22]. Bortezomib decreased TA via transcriptional as well as post-translation mechanisms. PKCα mediated the decrease in phosphorylation of telomerase by Bortezomib. Epoxomicin seem to be a much better drug in this sense compared to Bortezomib or MG-132.

The main limitation of our study is the absence of an epistatic experiment showing whether the inhibition of telomerase affected MM cells proliferation. Certainly, further studies are needed to elucidate the downstream phenotypic and molecular effects of the inhibition of telomerase in the setting of MM cells. 

Possibly, the combination of proteasome inhibitors that are clinically used with telomerase inhibitors such as Imetelstat will synergistically enhance their anticancer effects against MM cells, including those that developed resistance to proteasome inhibitors. Moreover, these combinations of telomerase inhibitors and proteasome inhibitors may also be relevant for the treatment of MM patients that are refractory to current modalities of anti-MM therapies.

## 4. Materials and Methods

### 4.1. Cell Culture

The experimental system was based on four MM cell lines: CAG, ARP-1, U266 and RPMI-8226. All cells were kindly provided to us by Prof. Lishner’s laboratory, Kfar-Saba, Israel. Cells were cultured in RPMI-1640 with 30% FBS, containing 2 mM L-Glutamine, 100 units/mL penicillin and 100µg/mL streptomycin. All ingredients were purchased from Biological Industries Beit Haemek, Israel.

### 4.2. Cell Viability and Proliferation Assays

Proliferation and viability were studied both by the WST-1 assay (Roche, Basel, Switzerland) and by Trypan Blue exclusion. WST-1 evaluates the activity of mitochondrial dehydrogenases that cleave the WST-1 reagent to a formazan dye and indicates cell number by measuring the absorbance at 450 nm in a microplate reader. To assess cell viability and proliferation, cells were cultured in 96-well plates for 24, 48, and 72 h and exposed to the relevant drugs.

### 4.3. Telomerase Activity Assay

Telomerase activity was assessed by the TRAP assay (TRAPeze kit, Millipore, Temecula, CA, USA) according to the manufacturer’s instructions in samples which were exposed to the IC_50_ dose of the drugs. Cells were lysed with ice-cold CHAPS lysis buffer, 50–100 ng of protein extracts were subjected to PCR in the presence of TS primer. The PCR products were separated on 12.5% PAGE, stained with Nucleic Acid Gel Stain (Lonza, Basel, Switzerland) and quantified by the Quantity One software in the Versa-Doc device. TA was calculated according to the following formula: TPG = [(X−B)/C]:[(r−B)/Cr × 100], where TPG is the total product generated, X signifies each sample signal, B is the gel background, C represents the 36 bp internal PCR control, r is the TSR8 quantification control.

### 4.4. Real-Time PCR

The expression of the hTERT gene was detected by Real Time PCR. Total RNA was extracted from cells using EZ-RNA Isolation Kit reagent (Biological Industries, Beit Haemek, Israel) and was reverse transcribed according to the manufacturer’s instructions of the High-Capacity cDNA Reverse Transcription Kits (Applied Biosystems, Foster City, CA, USA). The real-time quantitative RT-PCR is based on the TaqMan methodology, ABI PRISM 7000 Sequence Detection System (Applied Biosystems, CA. USA). hTERT gene expression was calculated relatively to the expression of the control gene HPRT-1.

### 4.5. Chromatin Immunoprecipitation Assay

The involvement of NF-κB, c-Myc and SP1 in the inhibitory effect of Bortezomib in MCL cells was assessed by the Chromatin ImmunoPrecipitation (ChIP) assay [23]. ChIP was performed with the EZ ChIP kit according to the manufacturer’s instructions (Upstate, Temecula, CA, USA) and as previously described [22]. The level of the DNA binding of SP1, c-Myc and NF-κB was evaluated relatively to the total DNA input of each sample that was not immunoprecipitated with any of the antibodies. The antibodies against SP1 and c-Myc were purchased from Millipore MA, USA. The antibodies against NF-κB were purchased from Abcam MA, USA. The relevant primers with the following sequences are as follows:

For the region of c-Myc and SP-1 binding site:Forward primer: AGTGGATTCGCGGGCACAGA;Reverse primer: TTCCCACGTGCGCAGCAGGAFor the region of NF-κB binding site:Forward primer: GCCTCCTAGCTCTGCAGTReverse primer: ACCCGAGGACGCATTGCTFor the binding site of WT1:Forward primer: TTTGCCCTAGTGGCAGAGAC Reverse primer: GCCGGAGGAAATTG

Due to the high GC content in the hTERT promoter region, real time PCR reactions included several additional temperature steps (in the polymerization stage) to ensure proper products formation. PCR conditions were as follows: 94 °C, 3 min, followed by 36 cycles of: 94 °C, 20 s; 58 °C, 30 s; 72 °C, 20 s; 76 °C, 20 s; 80 °C, 20 s; 84 °C, 20 s; and finally 72 °C, 5 min. Reaction mixture (25 μL) contained 2 μL DNA, 1.5 μL each of the primers and Quantitect SYBR Green master mix (Qiagen, Hilden, Germany). Reactions were performed in the ABI PRISM 7000 Sequence Detection System (Applied Biosystems, CA, USA). Values of each reaction were calculated versus the total input (were no antibody was used) in each case, respectively.

### 4.6. Immunoprecipitation and Western Blot Analysis

To determine the phosphorylated hTERT level, an immunoprecipitation assay was used. 500–1000 µg of protein was precipitated with 10 µg/mg anti-phosphoserine antibody (StressMarq Biosciences Inc. (Victoria, BC, Canada) or with 20 µg/mg anti-total hTERT antibody (Santa Cruz, Dallas, TX, USA). The protein-antibody complexes were mixed with 20 µg of protein G agarose beads for 16 h agitation in 4 °C. The immunoprecipitated lysates were washed with CHAPS lysis buffer and boiled for 3 min. for standard Western blot analysis, as described below. The primary anti-hTERT antibody for the immunobloting (1:500–1:1000) was purchased from Epitomics, Burlingame, CA, USA.

### 4.7. Statistical Analysis

A two-tailed One-sample Student *t* test with unequal variance and ANOVA one way were used to calculate the *p* values in SPSS for Windows version 11.5 software (SPSS, Inc., Chicago, IL, USA). All experiments were repeated independently at least three times. In all assays, *p* values <0.05 and 0.001 were considered statistically significant and highly significant, respectively.

## 5. Conclusions

The results of this study may contribute to future development of telomerase inhibition as a therapeutic modality in MM in combination with proteasome inhibitors that are clinically used. These combinations may also be relevant for the treatment of MM patients that are refractory to current modalities of anti-MM therapies.
